# Supplementary material for: Carbon and nitrogen signaling regulate FLOWERING LOCUS C and impact flowering time in Arabidopsis
Source: Plant Physiol. 2024 Nov 12;197(1):kiae594. doi: 10.1093/plphys/kiae594 (PMC11663559; doi:10.1093/plphys/kiae594)
Supplement: kiae594_Supplementary_Data [file kiae594_supplementary_data.docx]

Supplementary Data for

**Carbon and nitrogen signaling regulate *FLOWERING LOCUS C* and impact flowering time in Arabidopsis**

**Vladislav Gramma^1‡#^, Justyna Jadwiga Olas^1‡#^, Vasiliki Zacharaki^2#^, Jathish Ponnu^3^, Magdalena Musialak-Lange^1^**^‡^**, and Vanessa Wahl^1,4*^**

^1^Max Planck Institute of Molecular Plant Physiology, Department Metabolic Networks, Potsdam, Germany.

^2^Umeå Plant Science Centre, Department of Plant Physiology, Umeå University, Umeå, Sweden.

^3^Joseph Gottlieb Kölreuter Institute for Plant Sciences (JKIP), Karlsruhe Institute of Technology (KIT), Germany.

^4^The James Hutton Institute, Department of Cell and Molecular Sciences, Dundee, UK.

*Corresponding author/author responsible for distribution of materials

^#^These three authors (in alphabetical order) contributed equally.

^‡^**Current affiliations:** University of Applied Sciences Berlin (VG), Leibniz Institute of Vegetable and Ornamental Crop e. V, Großbeeren, Germany (JJO); Metasysx GmbH, Am Mühlenberg 11, Potsdam, Germany (MML)

**Author emails and ORCID**:

[Vladislav.Gramma@HTW-Berlin.de](mailto:Vladislav.Gramma@HTW-Berlin.de) (0000-0002-9890-3042)

[Olas@igzev.de](mailto:Olas-Apelt@igzev.de) (0000-0002-4311-6738)

[Vasiliki.Zacharaki@umu.se](mailto:Vasiliki.Zacharak@umu.se) (0000-0002-5543-2332)

[Jathish.Ponnu@kit.edu](mailto:Jathish.Ponnu@kit.edu) (0000-0002-3276-7068)

[musialak-lange@metasysx.com](mailto:musialak-lange@metasysx.com) (0000-0002-0388-8960)

[Vanessa.Wahl@hutton.ac.uk](mailto:Vanessa.Wahl@hutton.ac.uk) (0000-0001-7421-8801)

**Short title:** C and N signaling regulate *FLC*.

**Supplementary Figures**

**
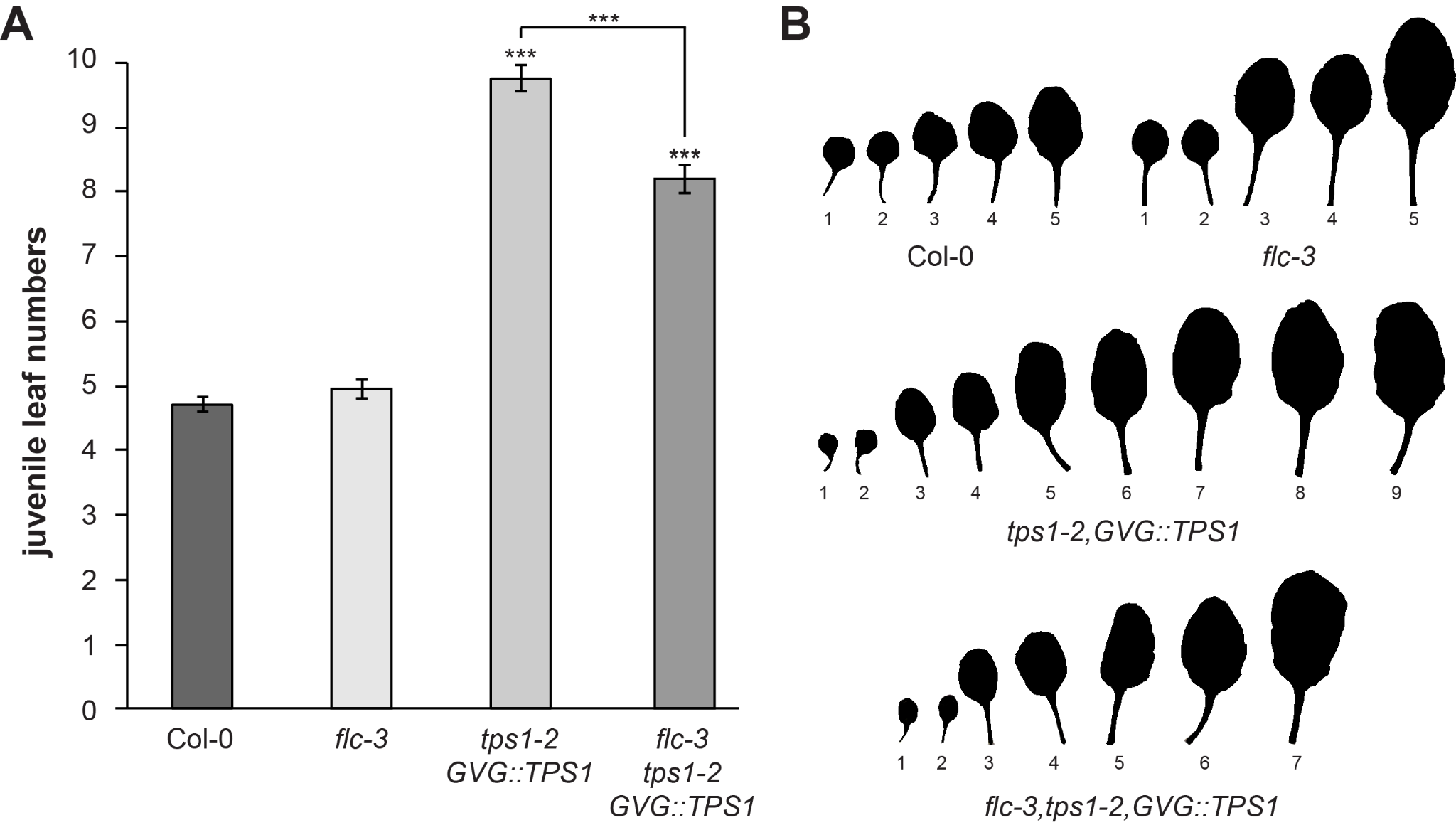
**

**Supplementary Figure S1**: ***flc-3* partially suppresses the delayed vegetative phase change phenotype of *tps1-2*,*GVG::TPS1* plants.** (**A**) Number of juvenile leaves recorded from wild-type Col-0 *flc-3*, *tps1-2,GVG::TPS1*, and *flc-3,tps1-2,GVG::TPS1* plants grown under long days (16h light/ 8h darkness). *n*= 20. (**B**) Leaf imprints of representative plants analyzed in (A). Data represents mean, error bars are standard deviations (s.d.), statistically significant difference compared to Col-0 wild-type (Student *t*-test, ****P*<0.001).


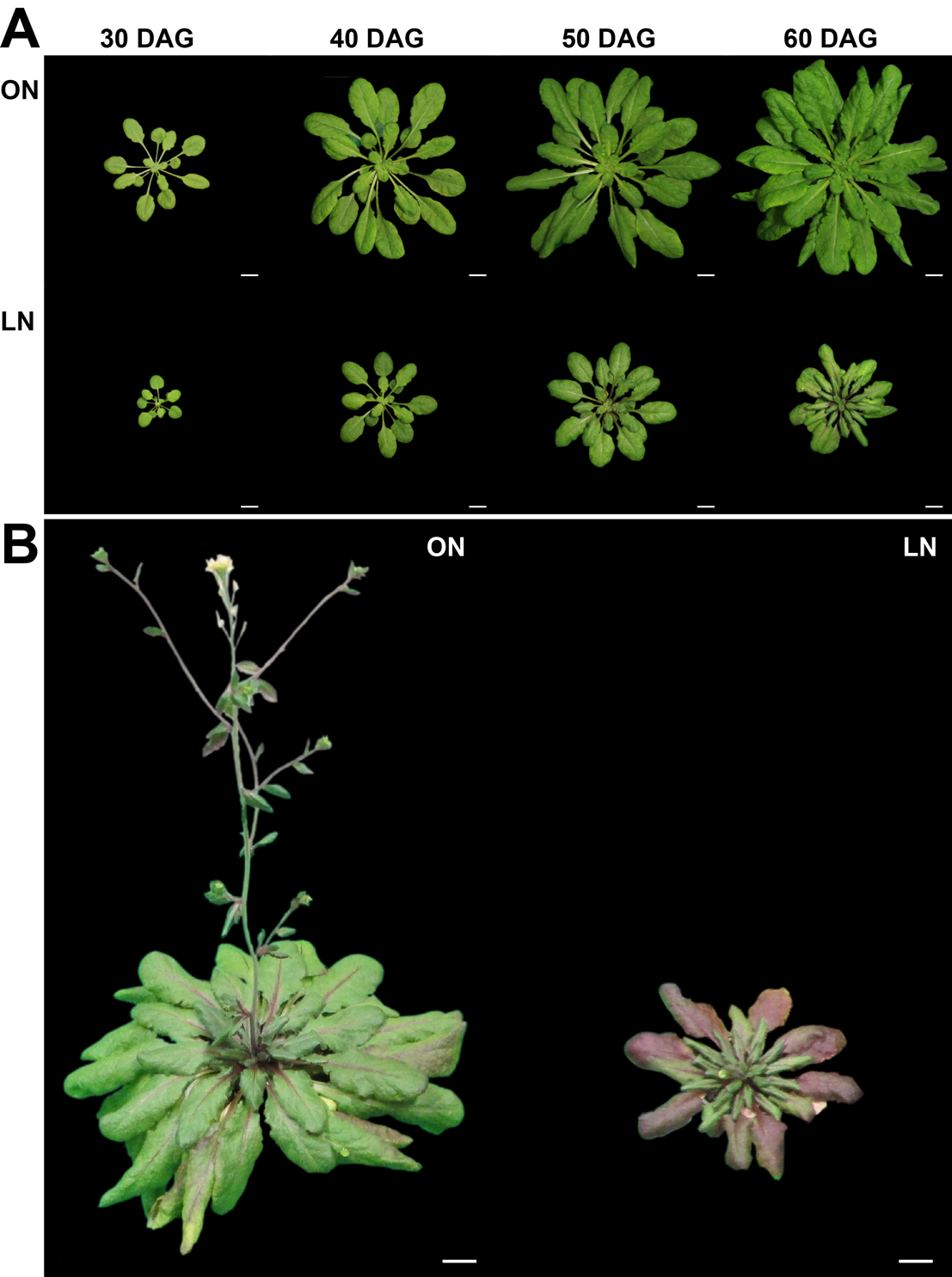


**Supplementary Figure S2**: **Phenotype in response to nitrogen (N) limitation of Col-0 plants continuously grown in short days (8h light, 16h dark).** (**A**) Representative photographs of Col-0 plants grown in optimal- and limited N content analyzed in Fig. 2A. (**B**) The same plants after bolting. Scale bars are 1cm. Images were digitally extracted for comparison.


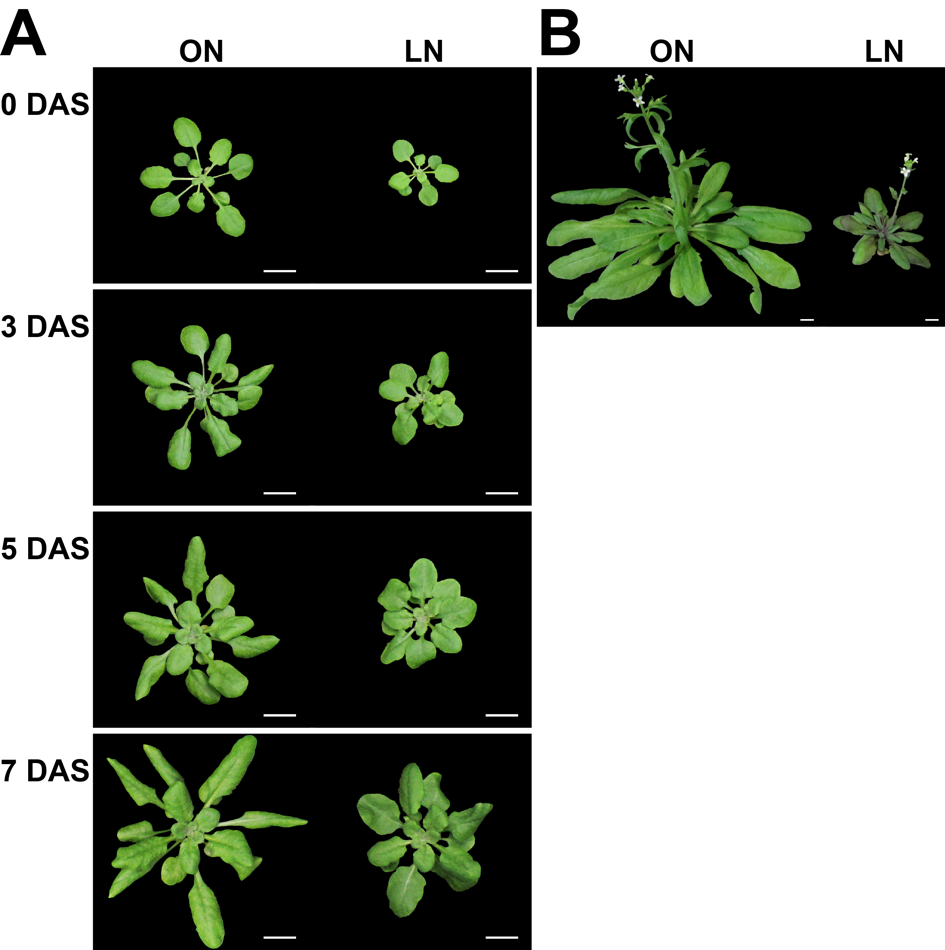


**Supplementary Figure S3**: **Phenotype in response to nitrogen (N) limitation of Col-0 plants transferred from short day (SD) to long day (LD) conditions.** (**A**) Representative photographs of plants analyzed in Fig. 2B,C. (**B**) The same plants after bolting. Scale bars are 1cm. Abbreviations: DAS (days after shift), ON (optimal nitrogen), LN (limited nitrogen). Images were digitally extracted for comparison.


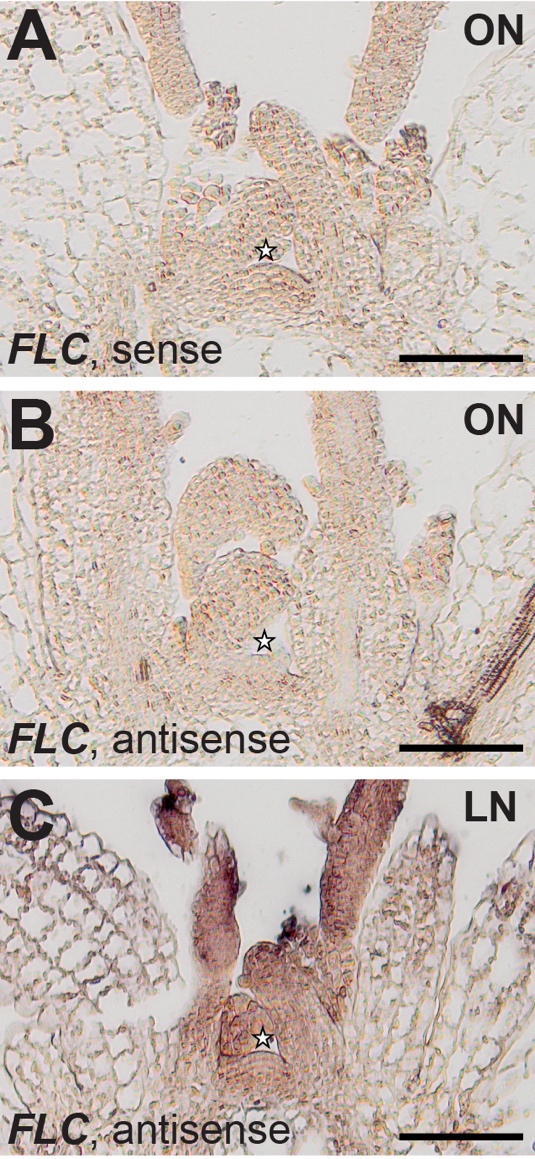


**Supplementary Figure S4**: **Additional pictures of RNA *in situ* hybridization with *FLOWERING LOCUS C* (*FLC*)-specific probes.** (**A**) Sense and (**B, C**) antisense probes hybridized on longitudinal sections through vegetative apices of Col-0 plants grown in optimal (ON) and limited nitrogen (LN) soils. Scale bars are 100µm. Star indicates summit of shoot apical meristem.


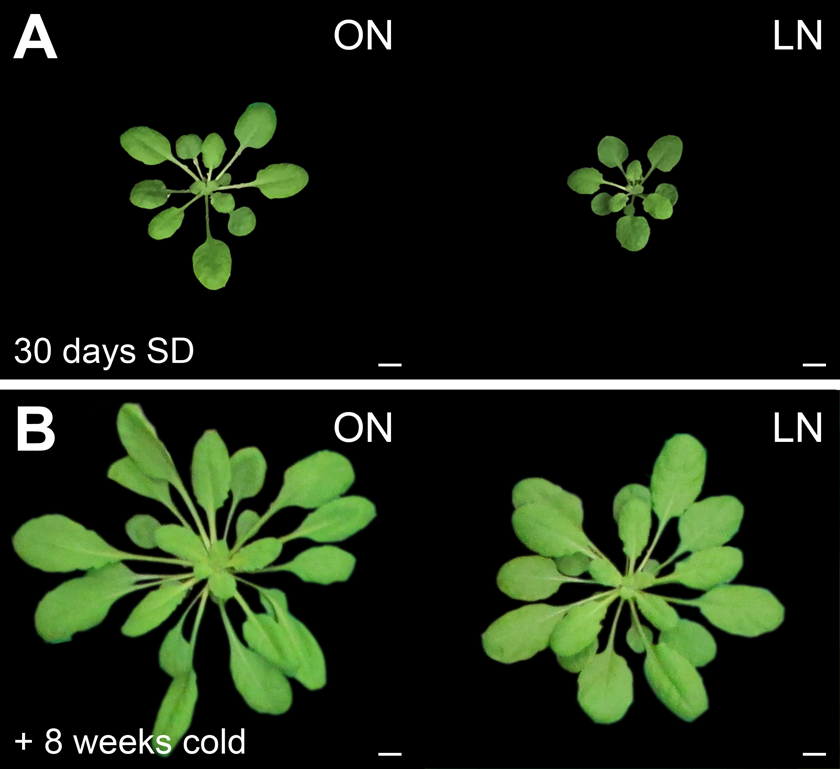


**Supplementary Figure S5**: **Phenotype of vernalized Col-0 plants in response to nitrogen (N) limitation.** (**A,B**) Representative photographs of plants analyzed in Fig. 3A. Before vernalization (**A**) and after vernalization (**B**). Scale bars are 1cm. Abbreviations: SD (short days), ON (optimal nitrogen), LN (limited nitrogen). Images were digitally extracted for comparison.

**
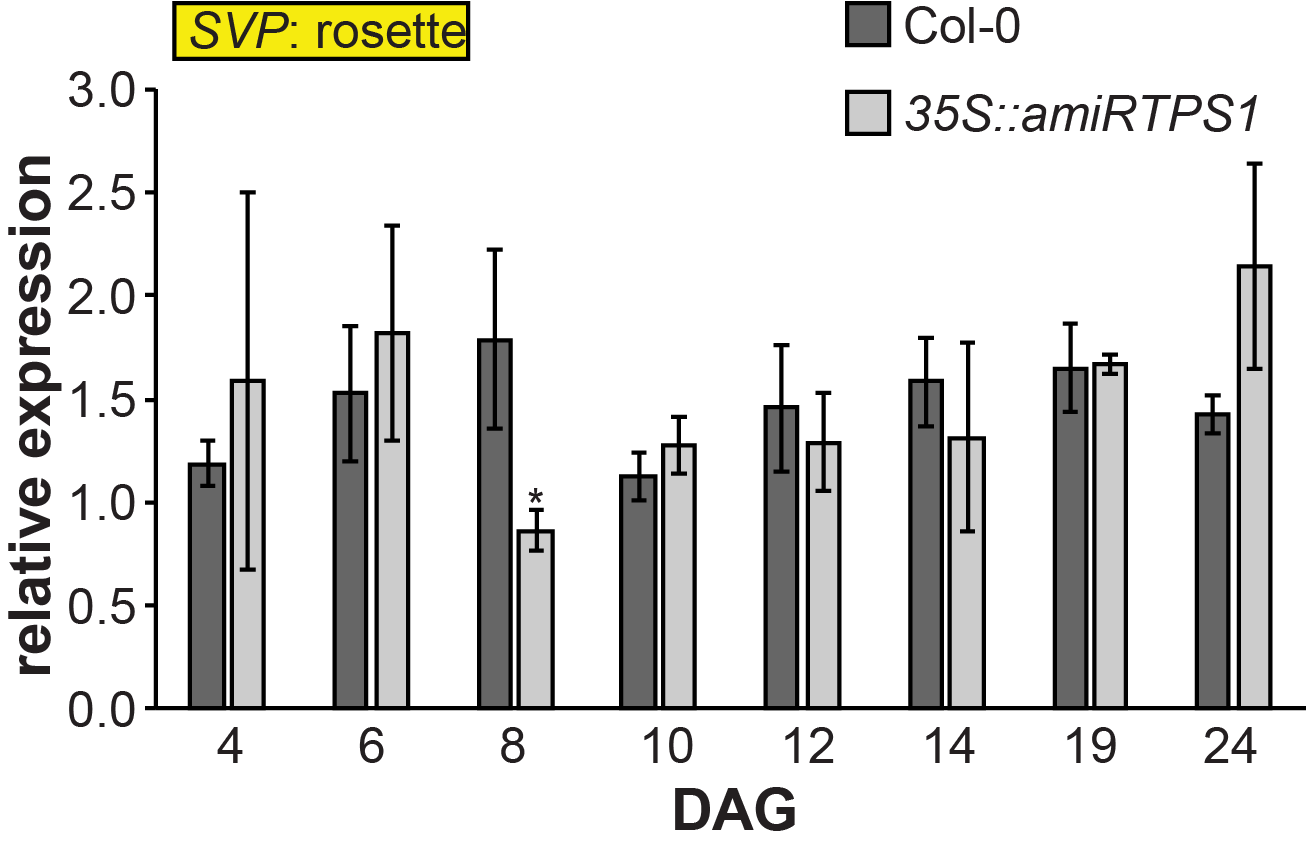
**

**Supplementary Figure S6**: ***SHORT VEGETATIVE PHASE* expression in *35S::amiRTPS1* plants.** Expression measured by RT-qPCR in rosettes of Col-0 and *35S::amiRTPS1* plants grown under long days (16h light/ 8h darkness). *n* = 4. Data represents mean, error bars are standard deviations (s.d.), significant difference compared to Col-0 wild-type (Student *t*-test, **P*<0.05). Abbreviations: days after germination (DAG).

**
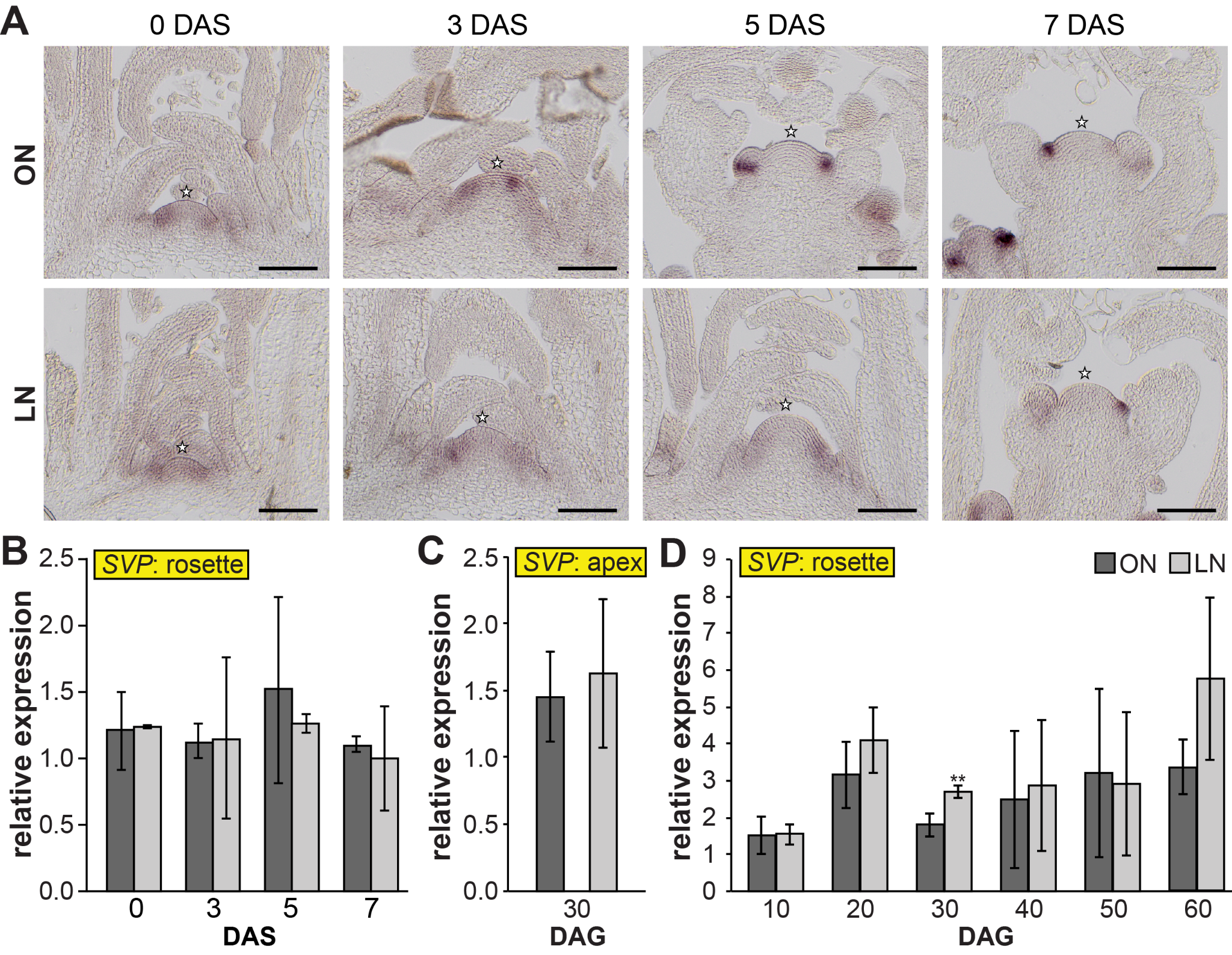
**

**Supplementary Figure S7**: ***SHORT VEGETATIVE PHASE* expression in response to nitrogen limitation. (A)** RNA *in situ* hybridization using *SHORT VEGETATIVE PHASE* specific probe on longitudinal sections through apices of Col-0 plants grown in optimal nitrogen (ON) and limited-nitrogen (LN) soils under short days (8h light/ 16h darkness) for the first 30 days and then transferred to long days (16h light/ 8h darkness) to initiate the floral transition for 3, 5, and 7 days (SD-LD shift). Scale bars are 100µm. **(B, C, D)** Expression measured by RT-qPCR in (**B**) apices of Col-0 plants grown in the SD-LD shift conditions, in (**C**) apices and (**D**) rosettes of plants continuously grown under short days. *n* = 3. Abbreviations: days after germination (DAG); days after shift (DAS). Data represents mean, error bars are standard deviations (s.d.), n=3, statistically significant difference between ON and LN (Student’s *t*-test, ***P*<0.01).


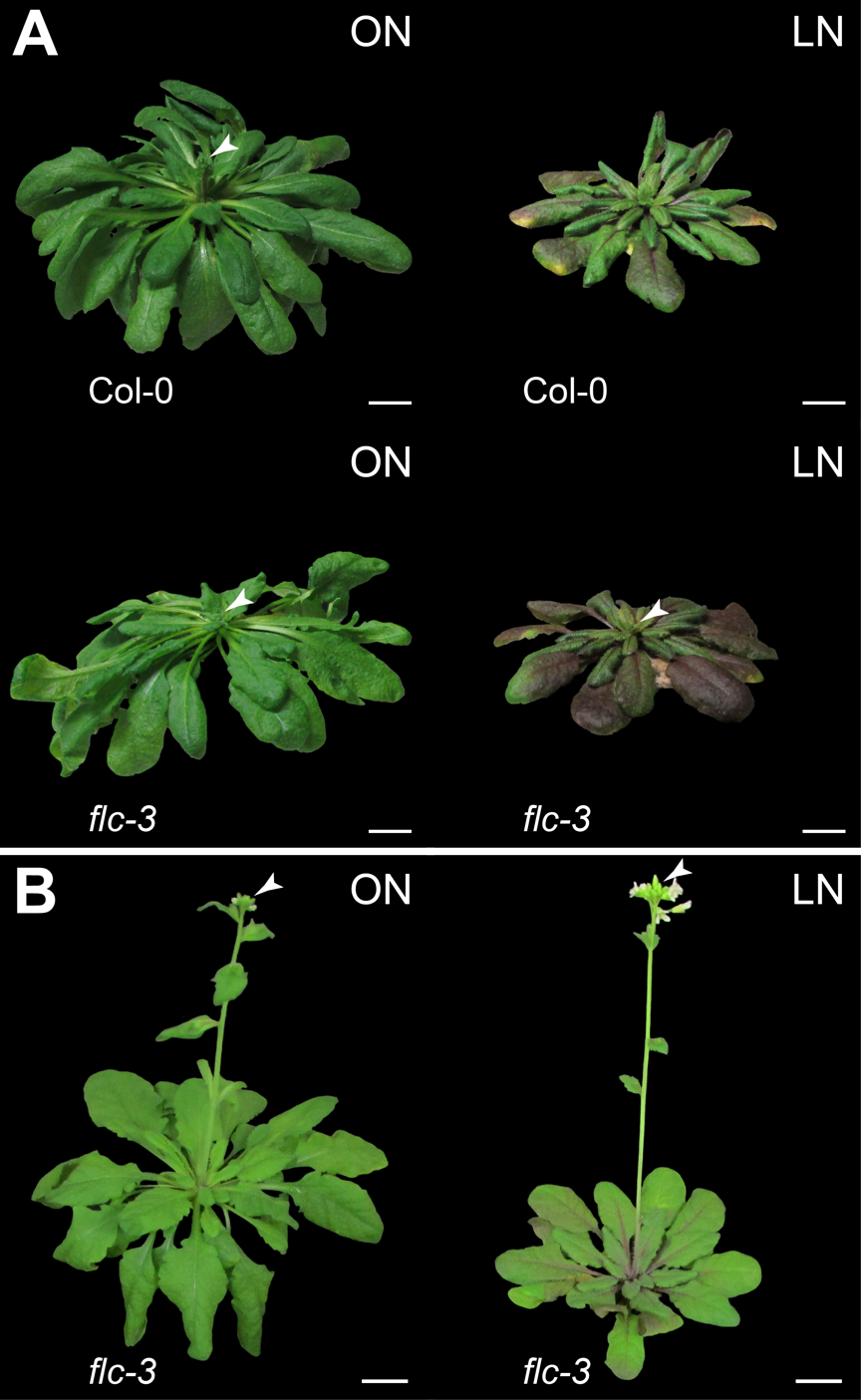


**Supplementary Figure S8**: **Phenotype of *flc-3* mutant plants in response to nitrogen (N) limitation.** (**A, B**) Representative photographs of plants analyzed in Fig. 3B. Col-0 and *flc-3* mutants at bolting (**A**) and *flc-3* after bolting (**B**). White arrow heads indicate inflorescence apices. Scale bars are 1cm. Abbreviations: ON (optimal nitrogen), LN (limited nitrogen). Images were digitally extracted for comparison.


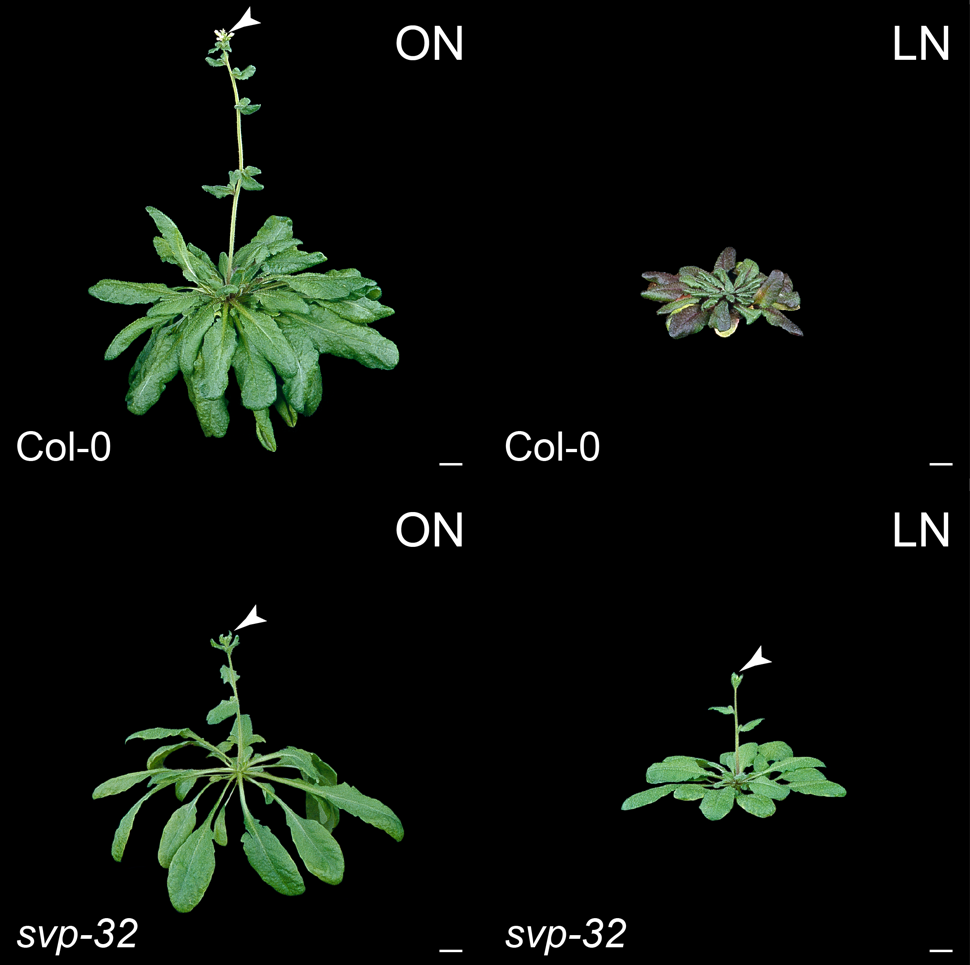


**Supplementary Figure S9**: **Phenotype of *svp-32* mutant plants in response to nitrogen (N) limitation.** Representative photographs of Col-0 and *svp-32* mutants analyzed in Fig. 3B. White arrow heads indicate inflorescence apices. Scale bars are 1cm. Abbreviations: ON (optimal nitrogen), LN (limited nitrogen). Images were digitally extracted for comparison.

**
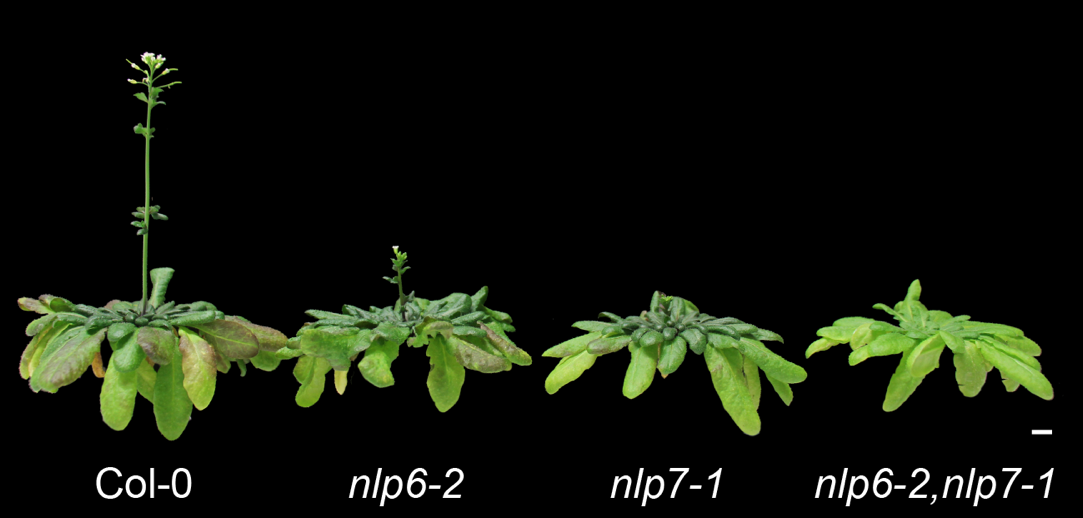
**

**Supplementary Figure S10**: **Phenotype of *nlp6* and *nlp7* mutant plants.** Representative pictures of the plants analyzed in Figure 4 at 90 days after germination. Scale bar is 1cm. Images were digitally extracted for comparison.

**
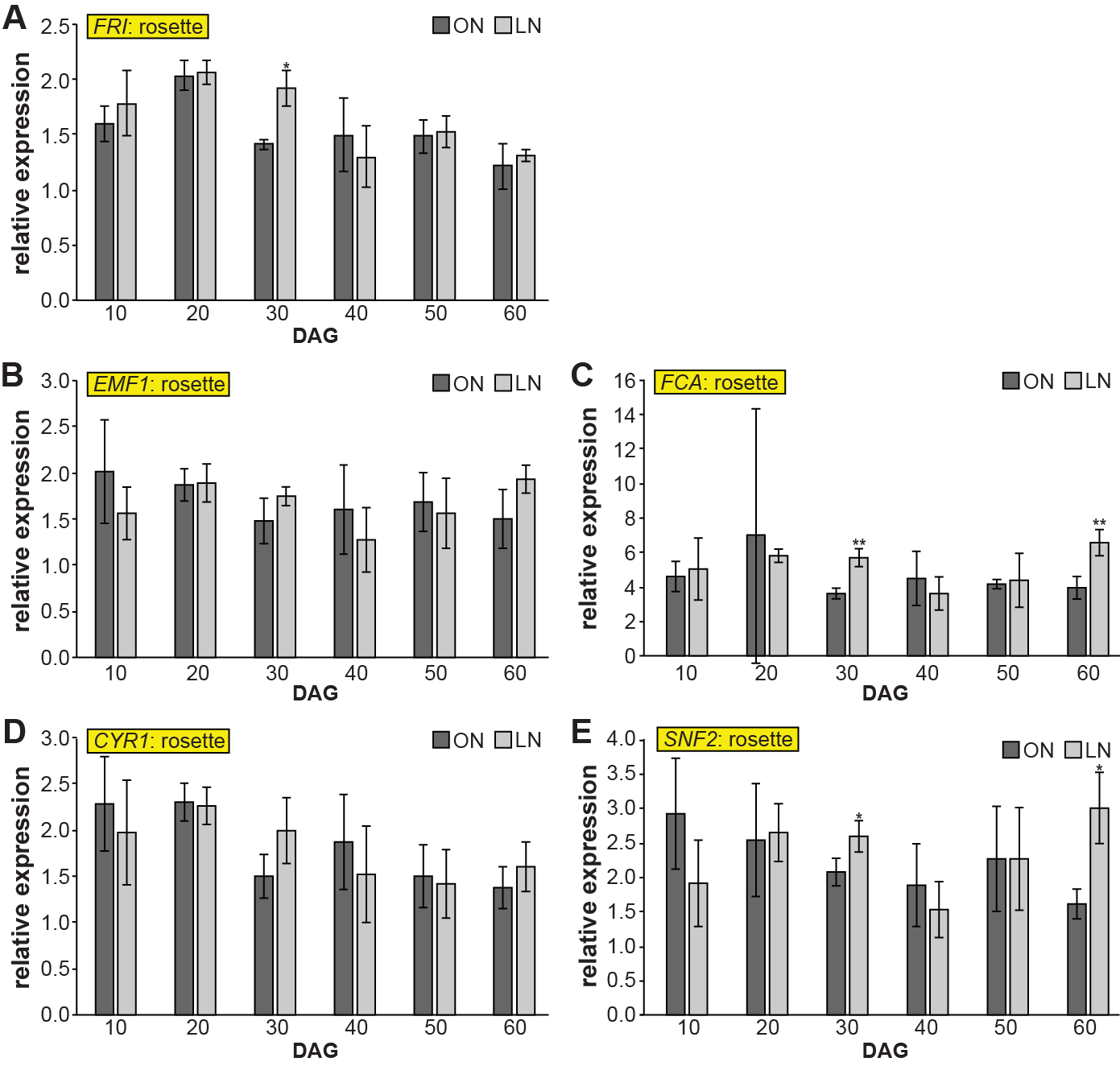
**

**Supplementary Figure S11**: **Regulators upstream of *FLOWERING LOCUS C* in response to nitrogen (N) limitation.** (**A**-**E**) Expression of Expression of (**A**) *FRI*, (**B**) *EMF1*, (**C**) *FCA*, (**D**) *CYR1/EMF2* and (**E**) *SNF2* measured by RT-qPCR in rosettes of Col-0 plants grown in optimal nitrogen (ON) and limited-nitrogen (LN) conditions under short days (16h light/ 8h dark). *n*= 3. Data represents mean, error bars are standard deviations (s.d.), statistically significant difference between ON and LN (Student’s *t*-test, **P*<0.05, ***P*<0.01). Abbreviations: days after germination (DAG).

**
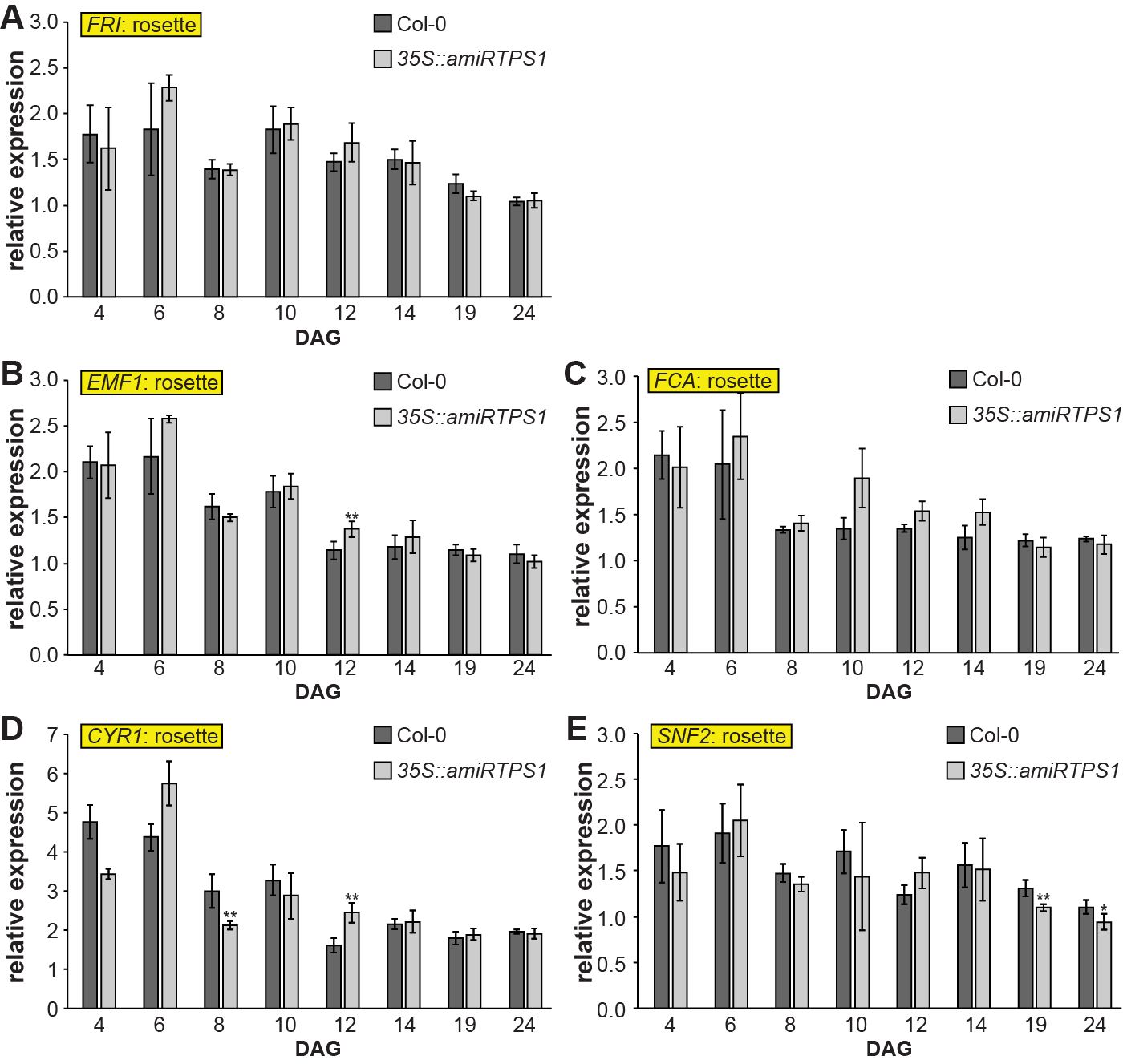
**

**Supplementary Figure S12**: **Regulators upstream of *FLOWERING LOCUS C* in *35S::amiRTPS1* plants.** (**A**-**E**) Expression of (A) *FRI*, (B) *EMF1*, (C) *FCA*, (D) *CYR1/EMF2* and (E) *SNF2* measured by RT-qPCR in rosettes of Col-0 and *35S::amiRTPS1* plants grown under long days (16h light/ 8h darkness). *N* = 4. Data represents mean, error bars are standard deviations (s.d.), statistically significant difference compared to Col-0 wild-type (Student *t*-test, **P*<0.05, ***P*<0.01). Abbreviations: days after germination (DAG).


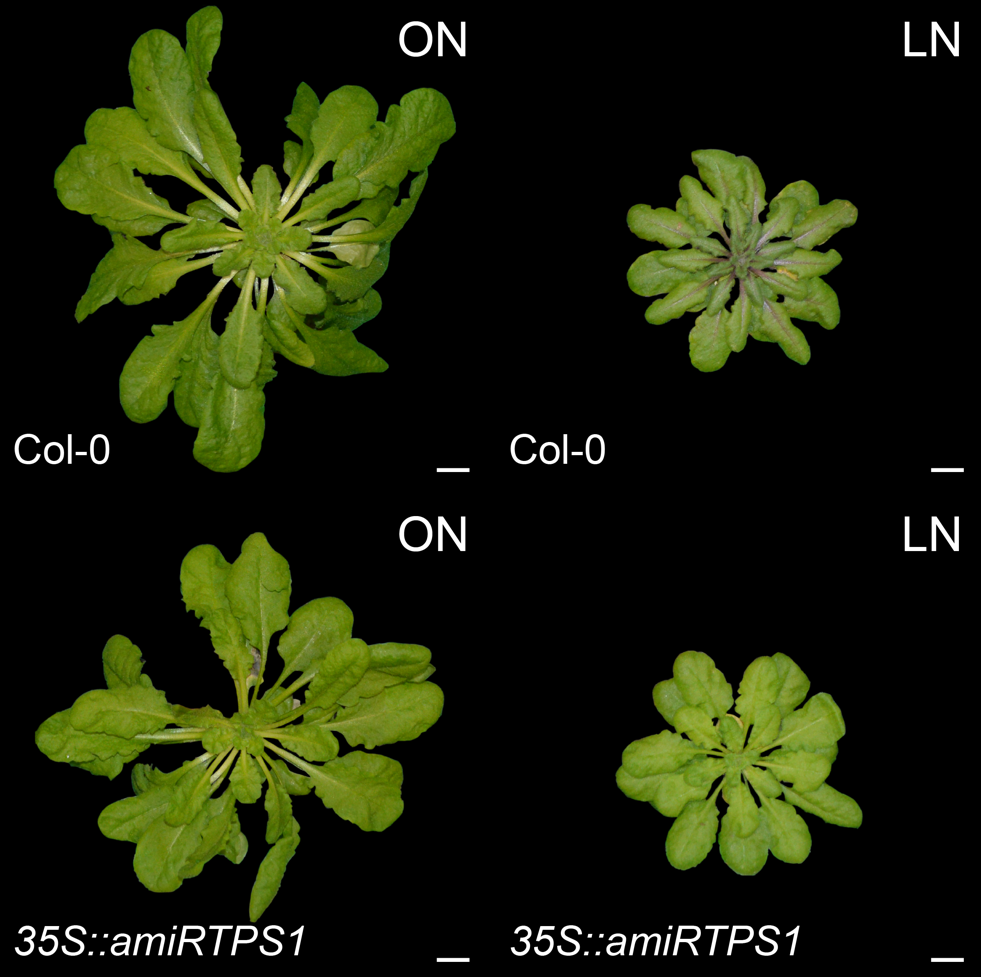


**Supplementary Figure S13**: **Phenotype of *35S::amiRTPS1* plants in response to nitrogen (N) limitation.** Representative photographs of Col-0 and *35S::amiRTPS1* plants analyzed in Fig. 5A. Scale bars are 1cm. Images were digitally extracted for comparison.


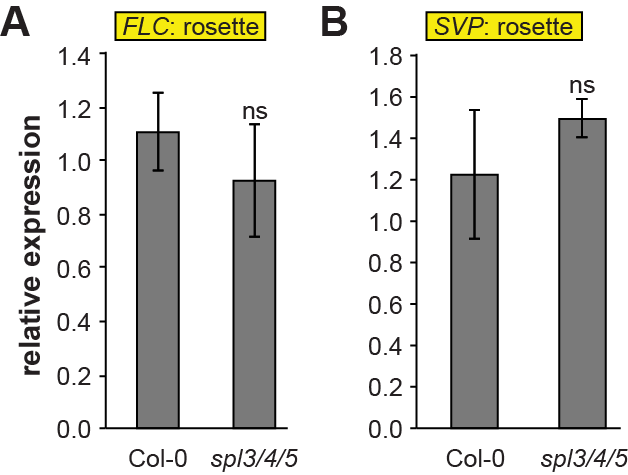


**Supplementary Figure S14**: ***FLOWERING LOCUS C* and *SHORT VEGETATIVE PHASE* expression in *spl3/4/5* mutant plants.** (**A, B**) Expression of (**A**) *FLOWERING LOCUS C* and (**B**) *SHORT VEGETATIVE PHASE* measured by RT-qPCR at 20 days after germination in rosettes of wild-type Col-0 and *spl3/4/5* mutant plants grown under long days (16h light/ 8h darkness). *n* = 3. Data represents mean, error bars are standard deviations (s.d.). Statistical significance of the difference between genotypes was calculated by Student’s *t*-test (ns – not significant).

**
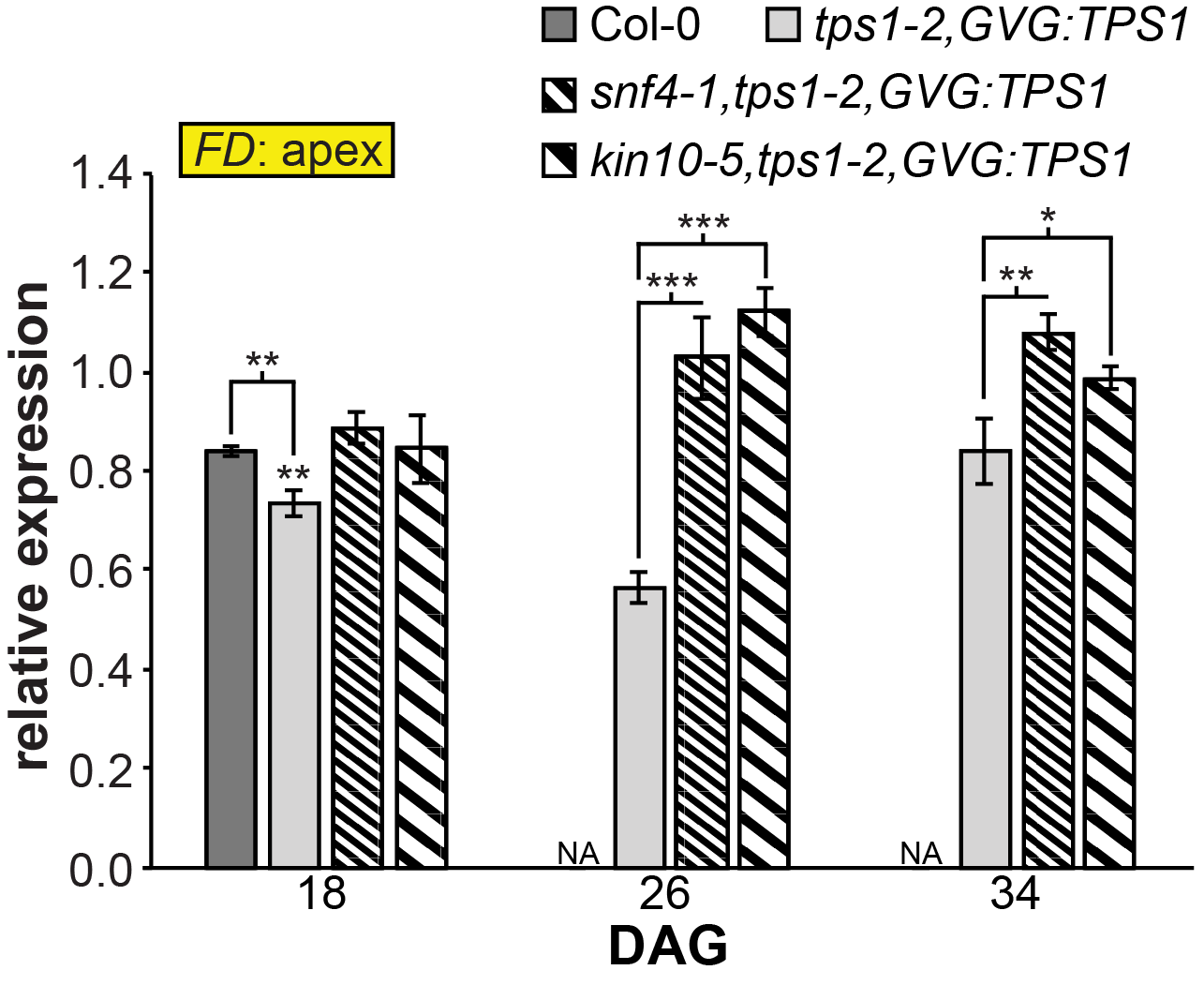
**

**Supplementary Figure S15**: ***FLOWERING LOCUS D* expression in *snrk1,tps1-2,GVG::TPS1* mutants.** RNA-seq data from apices of Col-0, *tps1-2,GVG::TPS1, kin10-5,tps1-2,GVG::TPS1* and *snf4-1,tps1-2,GVG::TPS1* apices. n=3. Data represents mean of VST expression, error bars are standard deviations (s.d.), statistically significant difference compared to Col-0 wild-type (Student *t*-test, **P*<0.05, ***P*<0.01, ****P*<0.001). Data obtained from available online RNA-seq dataset (Zacharaki et al., 2022).

**Supplementary Tables**

**Supplementary Table S1. Flowering time data of experiments described in this study.**

Abbreviations: DTF, days to flowering (bolting time) in bold as referred to in the main text; TLN, total leaf number; n, number of individual plants; (+/-) standard deviation, presence or absence of significance based on Student’s *t*-test calculated between plants grown on the ON and LN soils, respectively (+ : p-value < 0.05, - : p-value > 0.05).

| **Plant lines/Experiment** | **ON** | | | **LN** | | |
| --- | --- | --- | --- | --- | --- | --- |
|  | **DTF** | **TLN** | **n** | **DTF** | **TLN** | **n** |
| **Experiment 1 (long days) – Figure 1B** | | | | | | |
| Col-0 (wild type) | n.a. | **11.6 ± 0.9** | 20 | n.a. | n.a. | - |
| *flc-3* | n.a. | **9.5 ± 1.0** | 20 | n.a. | n.a. | - |
| *tps1-2, GVG::TPS1* | n.a. | **73.7 ± 4.6** | 20 | n.a. | n.a. | - |
| *flc-3, tps1-2, GVG::TPS1* | n.a. | **56.6 ± 5.1** | 20 | n.a. | n.a. | - |
| **Experiment 2 (short days/cold treated for 8 weeks) – Figure 3A** | | | | | | |
| Col-0 (wild type) | **89.3 ± 2.9** | 32.6 ± 2.3 | 17 | **87.9 ± 1.3^(-)^** | 30.5 ± 1.7 | 18 |
| *flc-3* | **91.2 ± 4.0** | 33.6 ± 4.3 | 18 | **92.4 ± 2.3^(-)^** | 35.0 ± 2.3 | 18 |
| **Experiment 3 (short days) – Figure 3B** | | | | | | |
| Col-0 (wild type) | **70.6 ± 4.5** | 68.3 ± 5.4 | 16 | **79.4 ± 2.4^(+)^** | 64.56 ± 2.0 | 16 |
| *flc-3* | **73.0 ± 5.1** | 65.3 ± 3.5 | 18 | **72.7 ± 2.9^(-)^** | 61.2 ± 3.2 | 18 |
| *svp-32* | **47.9 ± 3.1** | 33.9 ± 2.6 | 18 | **46.9 ± 2.5^(-)^** | 27.0 ± 2.2 | 18 |
| **Experiment 3b (short days) – repeat – Figure 3B** | | | | | | |
| Col-0 (wild type) | **66.2 ± 1.6** | 63.6 ± 2.3 | 16 | **81.1 ± 4.3^(+)^** | 62.1 ± 2.6 | 18 |
| *svp-32* | **44.1 ± 2.4** | 28.2 ± 2.1 | 19 | **46.3 ± 2.2^(-)^** | 28.1 ± 3.3 | 19 |
| **Experiment 3c (short days) – repeat – Figure 3B** | | | | | | |
| Col-0 (wild type) | **61.0 ± 2.4** | 61.4 ± 2.1 | 20 | **71.9 ± 2.1^(+)^** | 54.2 ± 2.0 | 17 |
| *flc-3* | **66.6 ± 3.0** | 59.5 ± 2.0 | 17 | **68.1 ± 2.2^(-)^** | 53.7 ± 2.3 | 18 |

**Supplementary Table S2. Vegetative phase change data of experiments described in this study (Figure S2).**

Abbreviations: JLN, juvenile leaf numbers in bold; n, number of individuals; +/- standard deviation, presence or absence of significance based on Student’s *t*-test (+ : p-value < 0.05).

| **Plant line** |  |  |
| --- | --- | --- |
|  | **JLN** | **n** |
| Col-0 (wild type) | **4.7 ± 0.5** | **20** |
| *tps1-2,GVG::TPS1* | **9.8 ± 0.9^(+)^** | **20** |
| *flc-3* | **5.0 ± 0.7^(+)^** | **20** |
| *flc-3,tps1-2,GVG::TPS1* | **8.2 ± 1.0^(+)^** | **20** |

**Supplementary Table S3. Putative nitrate responsive *cis*-elements (NREs) in regulators upstream of *FLOWERING LOCUS C*.**

| **Gene** | **Locus ID** | **NRE** |
| --- | --- | --- |
| NIR1 (consensus NRE) | AT2G15620 | tGaCCctT---(n)---AAGaG |
| FLC | At5g10140 | - |
| FRI | At4g00650 | TGACCgaTcatAAGAGAAGAG  TGACCaTTgatatatttatttctcaacagaaagAAGcG  aGACCacTaataagataccAAGtG  aGcCCaTTaccAAGAG |
| SUF4 | At1g30970 | - |
| ELF7 | At1g79730 | - |
| PIE1/SNF2 | At3g12810 | cGaCCtTTccttctacggcgcctaaaAAGgG |
| SEF | At5g37055 | - |
| VIN3 | At5g57380 | - |
| VRN1 | At3g18990 | - |
| VRN2 | At4g16845 | - |
| EMF1 | At5g11530 | TGaCCgTTttcagAAGAG |
| CYR1/EMF2 | At5g51230 | TGtCCaTTgctgcagctaaagtccatgagtgaggaaAAGtG |
| TFL2 | At5g17690 | - |
| FCA | At4g16280 | cGtCCaaTgggtcctaacggtggtgtgggaggagAAGgG |
| FVE | AT2G19520 | - |
| HUA2 | AT2G19520 | - |

**Supplementary Table S4. Oligonucleotides used in this study.**

| **Gene (AGI)** | **Oligo** | **Sequence (5’>3’)** | **Product lengths (bp)** |
| --- | --- | --- | --- |
| **Oligonucleotides used for RT-qPCR** | | | |
| ***TUB2***  At5g62690 | P-344  P-345 | GAGCCTTACAACGCTACTCTGTCTGTC  ACACCAGACATAGTAGCAGAAATCAAG | 167 |
| ***SAND***  At2g28390 | P-346  P-347 | AACTCTATGCAGCATTTGATCCACT  TGATTGCATATCTTTATCGCCATC | 61 |
| ***UBI10***  At4g05320 | P-348  P-349 | CACACTCCACTTGGTCTTGCGT  TGGTCTTTCCGGTGAGAGTCTTCA | 71 |
| ***PDF2***  At1g13320 | P-350  P-351 | TAACGTGGCCAAAATGATG  GTTCTCCACAACCGCTTGGT | 61 |
| ***FLC***  At5g10140 | P-402  P-403 | GAAGACCGAACTCATGTTGAAGCT  GCTCCCACATGATGATTATTCTCC | 114 |
| ***SOC1***  At2g45660 | P-532  P-533 | TTGAGCAGCTCAAGCAAAAGGA  TCCCCACTTTTCAGAGAGCTTCTC | 68 |
| ***SPL3***  At2g33810 | P-544  P-545 | GAGTTTGTCAGGTCGAGAGTTGTACC  GCAGACTTTGTGTCGTTTGTGGT | 74 |
| ***SPL4***  At1g53160 | P-546  P-547 | AATGGTCAGGTGGTGATGCAG  GCATAGGAAGTGTCATCTCTACCCTT | 61 |
| ***SPL5***  At3g15270 | P-548  P-549 | CAGCAGGTTTCATGAGCTACCAG  CAAAACTGTCACCAGAGATCTTCCTC | 107 |
| ***SVP***  At2g22540 | P-556  P-557 | CGGAGTCTATTACTAACGCCGGA  ATACGGTAAGCCGAGCCTAAGG | 89 |
| **Oligonucleotides used for genotyping** | | | |
| ***FLC***  [At5g10140](http://www.arabidopsis.org/servlets/TairObject?id=136002&type=locus) | P-0700  P-0701 | ATGGGAAGAAAAAAACTAGAAATC  CTAATTAAGTAGTGGGAGAGTCAC |  |

**Supplementary Reference**

**Zacharaki V, Ponnu J, Crepin N, Langenecker T, Hagmann J, Skorzinski N, Musialak-Lange M, Wahl V, Rolland F, Schmid M** (2022) Impaired KIN10 function restores developmental defects in the Arabidopsis trehalose 6-phosphate synthase1 (tps1) mutant. New Phytol **235:** 220-233
